# The Different Career Patterns of Two Pathbreaking Women Biologists at the Hebrew University of Jerusalem

**DOI:** 10.1007/s10739-025-09827-6

**Published:** 2025-09-17

**Authors:** Nurit Kirsh

**Affiliations:** The Biological Thought Program, The Open University of Israel, Raanana, Israel

**Keywords:** Women, Genetics, Botany, The Hebrew University of Jerusalem, Sticky floor, Glass ceiling

## Abstract

This article analyzes the different career patterns of Naomi Feinbrun, a botanist, and Elisheva Goldschmidt, a geneticist, both of whom began their PhD studies at the Hebrew University of Jerusalem in the 1930s, and were among the first women to receive professorships at the same university. Although Feinbrun experienced early career obstacles, she encountered less opposition while on the higher rungs of the academic ladder. In contrast, Goldschmidt, who operated according to a much more ambitious and competitive pattern than Feinbrun, did not suffer significant barriers at the beginning of her academic career, but encountered hurdles at a later stage. This article offers a close, comparative analysis of their careers, so that the distinctions and specificities of early and late career obstacles become clear. It argues that by paying attention to the intersectionality of gender with other categories like social and cultural backgrounds as well as personal characteristics, a complex picture emerges that moves beyond traditional gender-based sociological explanations that draw on the metaphors of “glass ceiling” and “sticky floor.” As the article shows, furthermore, even women scientists who belonged to similar disciplines in the same university and were active in the same time period, could face quite different challenges. Such comparative studies on the career patterns of women scientists might offer new possibilities for the many historical challenges faced by women scientists in various global contexts.

**Supplementary Information:**

The online version contains supplementary material available at 10.1007/s10739-025-09827-6.

## Introduction

Research on the history of women in science generally demonstrates two major trends. The first focuses on broad, quantitative studies that incorporate statistical data to analyze overarching patterns. A prime example of this type of research are Margaret Rossiter’s landmark studies, which provide a comprehensive look at the historical progression of women scientists in the United States (Rossiter 1982, 1995, 2012). The second trend delves into individual scientists’ biographies, which often reveals broader insights applicable to many women in science. Evelyn Fox Keller’s biography of Barbara McClintock is a notable example of this approach (Fox Keller 1983). Biographical research can also take a comparative approach, analyzing the lives of two or more figures to uncover insights that would be less apparent when focusing on just one person.

Although some philosophers of science debate the methodological legitimacy of using case studies to support general conclusions (Currie 2015; Mizrahi 2020), historians of science view the approach as a well established methodological practice. The present study, comparing two scientists’ careers, provides a productive framework for deploying conceptual tools, evaluating their explanatory adequacy in particular historical contexts, and formulating interpretations and hypotheses that may be further examined through subsequent case studies.

I have been investigating the scientific career of geneticist Elisheva Goldschmidt (1912–1970) associated with The Hebrew University of Jerusalem, for more than two decades (Kirsh 2004; Kirsh 2013). Recently, my growing familiarity with the career of another woman scientist who worked at the same institution, in the same time period, botanist Naomi Feinbrun (1900–1995), prompted me to compare their academic trajectories. The current study seeks to illuminate the similarities and differences in the professional experiences of these two patbreaking women scientists. In particular, it focuses on two pivotal junctures in an academic career: one’s first promotion to lecturer, and one’s advancement to full professorship.^1^ By analyzing the processes and circumstances that shaped these critical milestones, the article aims to contribute to a broader understanding of the structural and personal dynamics that have historically influenced women’s careers in science.

Botanist Naomi Feinbrun and geneticist Elisheva Goldschmidt both emigrated from Europe and began their PhD studies at the Hebrew University of Jerusalem during the 1930s (Heyn 1995; Kirsh 2004). By 1960, they were two of the first four women to attain a professorship at this university, and at a time when women were a small minority of the scientific faculty, not only locally but also worldwide. Feinbrun studied the flora of Israel for more than six decades and published dozens of articles and several analytical botanical books. She was an Israel Prize laureate for her unique contribution to “Land of Israel” studies.^2^ Goldschmidt also made her mark on Israel’s science. She established and headed the genetics studies circle at The Hebrew University of Jerusalem (later to become an independent department), and is considered the “founding mother” of genetics research in Israel. She founded and headed the Israeli genetic society and wrote the entry for “genetics” for the *Hebrew Encyclopedia*.

Despite noteworthy similarities, there is one significant difference between the two women—Feinbrun and Goldschmidt came of age in different historical and geographical contexts. Goldschmidt was born and raised in Germany, while Feinbrun, twelve years her senior, grew up in Eastern Europe. The potential impact of these early formative environments will be examined in a later section of this article. Furthermore, although both scientists began their academic journeys in classical biological sub-disciplines, their professional trajectories ultimately diverged. Feinbrun remained dedicated to botany throughout her career, whereas Goldschmidt, initially trained in zoology, later specialized in genetics. Entering a relatively new academic field gave Goldschmidt a strategic advantage and contributed to her becoming Israel’s first academic geneticist.

As women scientists, Feinbrun and Goldschmidt encountered obstacles which, although they did not block the path to their academic promotion, clearly slowed it down. Yet the timing of the obstacles and their influence on their academic careers were different. Feinbrun mostly encountered hurdles during the early stages of her career, a phenomenon known as the “sticky floor,” while Goldschmidt enjoyed a period of speedy academic progress before she was halted at the last stage of the academic ladder by a “glass ceiling,” which she eventually overcame.

The term “glass ceiling” was coined in 1978 by Marilyn Loden, a human resources professional in the telecommunication industry, to characterize the barriers, mostly invisible, that prevented women from rising in their careers beyond a certain level in a hierarchy.^3^ Sixteen years later, sociologist Catherine White Berheide evoked the “sticky floor” metaphor to describe discriminatory patterns that kept workers, mainly women, in the lower ranks of the job scale, with lower pay and less power and prestige (Harlan and White Berheide 1994). The “glass ceiling” and “sticky floor” metaphors (as well as the “leaky pipeline”), were coined to explain the failure of 1970s affirmative action legislation in the United States to secure equal opportunities of academic promotion for women and minorities. The context dealt with in this article is different, referring to an entirely different region of the world, so that the applicability of these metaphors might be questionable. In addition, using sociological terms potentially deflects attention from the agency of those responsible for women’s under-representation in science and reduces them to mere structural features. Hence, in the following pages I adopt instead the terms “early career obstacles” and “late career obstacles,” as opposed to relying on the concepts of “sticky floors” and “glass ceilings,” to explain the divergent patterns observable in the careers of these two women scientists. While my analysis focuses on two individual cases, the patterns of early and late career obstacles may reflect broader dynamics experienced by other women in science.

## Naomi Feinbrun

Born to a Zionist Jewish family in Moscow in 1900, Naomi Feinbrun began her academic studies in 1918 at the Faculty of Natural Sciences at Moscow University.^4^ When her family moved to Bessarabia in 1920, Feinbrun continued her studies at the University of Romania, in Cluj, Transylvania, where she received her first degree in botany in 1923. She then taught natural sciences for a while at the Jewish girls’ high school in Kishinev.^5^

In 1924, Feinbrun immigrated to Mandatory Palestine with her parents and siblings, and started working as a schoolteacher in the Jezreel Valley, a large fertile plain between the Jordan Valley and the coastal plain. In 1925, on a study tour for teachers of natural sciences to the Tavor Mountain, she met someone who would change her life—the botanist Alexander Eig (1894–1938), who guided the tour and instilled in her the enthusiasm to be a researcher. He remained her mentor and colleague until his death in 1938 (Ashbel 1991). Following the meeting with Eig, Feinbrun was accepted as a guest researcher and a few months later was accepted as a part-time researcher by the Institute of Agriculture and Natural History in Tel-Aviv. While working at the Institute, Feinbrun, who did not know English, learned the language, mostly by using the book *Flora of Syria*,* Palestine and Sinai* (Ashbel 1991; Post 1896).

When the Hebrew University of Jerusalem was founded in April 1925, the Institute in Tel-Aviv became part of the university and its name changed to the Systematic Botany Branch. Most of the Hebrew University’s faculty at its founding were German-born or educated, and this trend grew more pronounced with the Nazi rise to power. The campus on Mount Scopus reflected this cultural and intellectual orientation: the buildings were designed in the German architectural style, laboratory equipment was imported from Germany, and German was the primary—and for several years the only—foreign language taught at the university (Katz and Heyd 1997). Emigrant Jewish scientists from Germany brought with them the academic model in which they had been trained, characterized by its emphasis on uniformity in teaching and research, and a strict hierarchical faculty structure. This model had a decisive influence on the development of the natural sciences at the university, particularly in biology, where much of the early research focused primarily on field-based, observational studies. A gradual shift began to occur after World War II, when American academic influence became more pronounced. In the life sciences, this transition marked a turn from observational approaches conducted mainly in the field to laboratory-based experimental research.

In 1929, Feinbrun began working in Jerusalem as an untenured assistant at the university, together with Alexander Eig and Michael Zohary (1898–1983).^6^ Together, the three botanists published the first full analytical flora in Hebrew in 1931. In 1936, Eig established the *Palestine Journal of Botany: Jerusalem Series* in which Feinbrun and her colleagues published their work (Ashbel 1991).^7^

Budgetary problems and the distance from the scientific community plagued research at The Hebrew University in this period. It was especially isolated during World War II. The land itself, however, offered some compensation. Bridging Europe, Africa, and Asia, the area was rife with plant and animal life that were transitional links between species on these continents. Consequently, many field trips were conducted not only within the official borders of Mandatory Palestine but also across the wider region, in order to study the local fauna and flora. Systematics research was based on the European scientific tradition, and on the colonial tradition of creating collections for museums of natural history; yet it was also influenced by the national atmosphere of an emotional relationship to the landscape of the “ancient homeland” and its neighboring countries (Kirsh 2022).

In 1933, Feinbrun joined a delegation of seven Hebrew University scientists who were invited to Iraq by the Iraqi Ministry of Agriculture. Their main purpose was to conduct a survey of the forests of Kurdistan, prepare an inventory of trees, and present a proposal for afforestation and for preserving the forests (Evenari 1985). Other research expeditions she took part in during the 1930s and early 1940s were to Transjordan, the Sinai Peninsula, Lebanon, Cyprus, and the eastern desert in Egypt, territories then variously under British or French control or influence.^8^

In its early years, the Hebrew University operated as a research facility without formal teaching courses. Teaching in the sciences began in the early 1930s and genetics was one of the six major subjects proposed in the curriculum of the Department of Botany; Naomi Feinbrun was selected to teach this subject (Kirsh 2009). In 1931 Feinbrun traveled to Germany for six months to expand her expertise. During her stay, she worked at two institutes within the Kaiser Wilhelm network: the *Kaiser-Wilhelm-Institut für Züchtungsforschung* (Institute for Plant Breeding Research), directed by Erwin Baur in Müncheberg, and the *Kaiser-Wilhelm-Institut für Vererbungsforschung* (Institute for Heredity Research) in Berlin-Dahlem. In 1935, Feinbrun also spent three months at Professor Alexandre Guilliermond’s laboratory at Sorbonne University, Paris.^9^ Upon her return to Jerusalem she started to teach genetics and cytology, and until 1948 was the only one teaching a genetics course at the Hebrew University (Kirsh 2009, p. 169).

While teaching these courses, Feinbrun worked on her doctoral dissertation, titled “A Monographic Study of the Genus *Bellevalia*,*”* with Eig as her supervisor, studying the number and form of chromosomes and using them in the systematic classification of this plant genus. She received her PhD in 1938, but even before that Eig recommended that she be promoted to the rank of a full-fledged assistant (later this rank was named instructor).^10^ Following Eig’s untimely death, other members of the botany department repeated the request and explained in a four page letter why Feinbrun had to be promoted.^11^ Six years later, Michael Evenari wrote to the university’s management: “There is no justification for further delaying Miss Feinbrun’s appointment to an instructor, a position she has actually been holding for eight years or more.”^12^

At the end of 1944, the dean of the Mathematics and Natural Sciences Faculty wrote to the University’s rector:

I know that Miss Feinbrun is absolutely qualified to play the role of a certified assistant and in fact has been doing this work for several years […] Her appointment as an instructor would therefore constitute a suitable status for the situation that actually exists.^13^

Five months later, the members of the committee for the appointment of an instructor in botany claimed that Feinbrun,

Should have been given the rank of instructor a long time ago […] and this is a completely abnormal situation and the only case in the university where independent teaching was assigned in basic subjects! to an employee with a rank lower than instructor and the instructor rank was still not given […] Miss Feinbrun has been deprived for a long time and there is no justification for the continuation of the deprivation.^14^

Feinbrun was finally promoted to the rank of instructor in 1945, and it took an additional six years until she was promoted from an instructor to a lecturer.

As a researcher, Feinbrun devoted her full attention to the study of local and Middle Eastern species, mostly grown in her experimental plots and investigated cytotaxonomically. She spent the year 1953 on sabbatical conducting research in the Kew Gardens herbarium near London and in the herbaria of Edinburgh and Geneva.^15^

Feinbrun finally became an associate professor in 1960. At the time, there were only three other women with the same rank at the Hebrew University—and not even one at the rank of full professor. The other three women professors were geneticist Elisheva Goldschmidt, botanist Tscharna Rayss and pathologist Hanna Rozin, all in various fields of biology and medical sciences. A year later parasitologist Aviva Zuckerman was also promoted to the rank of associate professor.^16^ The concentration of these careers in the biomedical sciences reflected the broader pattern of greater gender equity within these disciplines as compared to the physical sciences, as Rossiter has shown in her study of women scientists in the United States (Rossiter 1997).

Feinbrun did not stop working, not as an octogenarian, nor in her early nineties. The two volumes of *Flora of Palestine*, one of her most important works, were published in 1987, for which she won a gold medal from “Optima,” the international organization of Mediterranean botanists (Ashbel 1991). In 1991 a new and updated analytical flora, co-written with Avinoam Danin, appeared in print (Feinbrun-Dothan and Danin 1998).

In 1991, at the age of ninety-one, Feinbrun received the prestigious Israel Prize, conferred every Independence Day on citizens who have made outstanding contributions to society (Barak 2018). She won the prize for her unique contribution to “Land of Israel” studies. She passed away four years later, shortly before her ninety-fifth birthday. Her memory is still alive in plant names given in her honor by colleagues in Israel and abroad, among them *Astragalus feinbrniae* and *Bellevalia feinbrniae* in 1970, and *Colchicum feinbrniae* in 1992 (Heyn 1995).

## Elisheva Goldschmidt

Elisheva Goldschmidt was born as Elisabeth Wexler to an Orthodox Jewish family in Frankfurt-am-Main in 1912. She began to study medicine in 1932 at Frankfurt University, but her studies came to an abrupt end only one year later, when the Nazis came to power (Zneimer 1978). She chose to move to London, where she had relatives. She was accepted to the University of London, but for zoology and not to medical school, and thus her dream of becoming a physician ended.^17^ She graduated *cum laude*, and married Joseph Goldschmidt who had been her boyfriend since their days together in a youth movement in Frankfurt. She followed Joseph to Jerusalem, where he was already working as a school principal.^18^

Goldschmidt enrolled in a doctoral program at The Hebrew University and studied the giant chromosomes of *Chironomidae* mosquitoes found locally, under the supervision of zoologist George Haas (Goldschmidt 1942).^19^ In 1944, two years after receiving her Doctorate, Goldschmidt was accepted as an untenured assistant at the Department of Zoology. Her adjustment to the country was relatively easier than that of other Jewish immigrants from Germany who arrived in Mandatory Palestine during the 1930s. About half of these immigrants had to retrain in new occupations, and sometimes the change was quite extreme (Getter 1979). Several factors helped facilitate Elisabeth Goldschmidt’s adjustment to her new homeland. Having grown up in an Orthodox family she knew some Hebrew, and because of her natural ability to acquire languages, she learned the language quickly and well (Wahrman 1972).^20^ Furthermore, most German immigrants did not speak English, a language in which Goldschmidt was fluent (Wormann 1970). Finally, Goldschmidt had arrived in Jerusalem after her fiance, Joseph, who had already lived and worked there for a year.

Goldschmidt’s parents arrived in Jerusalem in 1946, around the time her younger daughter was born, and lived with the Goldschmidts and their two children. The couple had daily household help, but it was the presence of her parents that enabled Goldschmidt to pursue her scientific work. Her mother was instrumental in running the household and raising the children, which allowed Goldschmidt to spend long hours in the lab. Furthermore, it enabled her to view her desire for an academic career as a legitimate one. Thus, after the birth of her daughter, Goldschmidt did not take a true maternity leave, something she had done after the birth of her son, a few years earlier.^21^

Goldschmidt’s case presents a unique model of an extended family framework with a mother supporting and helping her scientist daughter. Many female scientists were forced to give up the conventional family model in order to fulfill their professional ambitions. Some of those who married stopped or greatly reduced their scientific work upon their marriage and returned to scientific activity only if, and when, they became widows. Others married and continued their scientific activities but did not have children (Abir-Am and Outram 1989). There were also women who functioned as wives, mothers and scientists and shared both the burden of the family and joint scientific research with their husband (Pycior, Slack and Abir-Am 1996; Lykknes, Opitz, and Van Tiggelen 2012). Of the first five women who made it to professorship at The Hebrew University, Goldschmidt was the only one who was married and the only one who had children. Researchers have shown that it is not marriage and motherhood that hinder women’s advancement in the academic world; to some degree there is even a positive correlation between family life and women’s success in academia (Frank-Fox 2005; Toren 2000, 87–105). However, there is often a price to be paid when both realms of life are successful – fatigue, frustration, and feelings of guilt.

During the war for Israel’s independence (1948–1949), The Hebrew University was cut off from the city of Jerusalem, and Goldschmidt moved her scientific work into her home. Tuvia Kushnir, her most brilliant student, was killed in the war and his young widow, Aviva, continued his research at Goldschmidt’s home, spending hours using the microscope. Although the transition from lab to home was forced upon her by war, it also represented a kind of retirement from the public, masculine arena, to the domestic, feminine one. This replayed a common pattern for women scientists working in other contexts. Anne May Lutz for example, was another woman geneticist who faced tribulations in her career and returned to the safer domestic sphere working out of her family home in Indiana for a number of years (Richmond 2010). Rita Levi-Montalcini, who specialized in the study of the nervous system, also carried out work from home—after the expulsion of Jewish students from Italian universities during World War II, she set up a laboratory in her bedroom (Levi-Montalcini 1988; see also Opitz et al. 2016).

When the war was over, Goldschmidt was still an untenured assistant at the Hebrew University. She was, however, already seen as having great promise in the field of genetics.^22^ The first academic position in genetics in Israel was created at The Hebrew University of Jerusalem in 1949. The faculty chose not to advertise the position, because they felt that Elisabeth Goldschmidt was the only suitable candidate. It was only after she became an instructor, in 1949, that she enjoyed the academic freedom to teach courses in addition to those in the established curriculum. Thus, she gave a series of elective lectures during the summer semester on advances in genetics, the first time she actually taught genetics.^23^ Together with the decision to appoint Goldschmidt as instructor, the university applied to the American Friends of the Hebrew University for a one-year study grant to conduct research in the United States.^24^ Goldschmidt received a grant from the American Association of University Women (AAUW), but she worried about the long separation from her family. When her male colleagues went overseas, they were accompanied by their wives,^25^ but Goldschmidt was faced with going without her husband and children. Despite her hesitancy, Goldschmidt decided to go, largely owing to her husband’s strong encouragement and the help they received from her mother in taking care of the children. She left her three-and-a half-year-old daughter and twelve-year-old son with her husband and her parents in Jerusalem. The year-long separation may have been difficult on the family, but the opportunity was extremely significant for the development of Goldschmidt’s scientific career.

Goldschmidt spent the first part of the year working with Theodosius Dobzhansky, one of the most prominent biologists of the 20th century, in his lab at Columbia University in New York. Dobzhansky was interested in the possibility that Goldschmidt would study the *Drosophila* population of Israel, an area that had not yet been studied. Following his advice she collected flies in Israel, especially from habitats remote from human settlements, and studied them upon arrival in the United States.^26^ She spent the second part of the year at Berkeley in Curt Stern’s lab, one of the world leading geneticists at that time.^27^ Following the publication of his 1949 book *Principles of Human Genetics* and its subsequent translation into several languages, Stern became a world-renowned authority on human genetics. The two researchers with whom Goldschmidt worked that year, Dobzhansky and Stern, inspired her approach to genetics. Both were among the most influential leaders in the field. Dobzhansky was the President of the Genetics Society of America in 1941, and Stern held the position in 1950.

Goldschmidt first encountered professional geneticists and genetics research during this trip to the United States. Biologists at the Hebrew University had a theoretical knowledge of genetics, and some used cytological tests on chromosomes as a research tool, but none had conducted actual genetic research.^28^ The time spent with leading geneticists, and the acquaintance with their research problems and methods opened a new and fascinating world for her. When Goldschmidt returned to Israel, she maintained contact with Dobzhansky and Stern and consulted with them occasionally (Goldschmidt 1952; Goldschmidt et al. 1955). Other than her first trip, no other overseas trip that she took lasted more than six months, but she managed to make the most out of each trip and visit.

While maintaining her interest in *Drosophila* populations and karyotypes, Goldschmidt became more involved in human genetics. In the summer of 1953 Goldschmidt visited eugenics institutes in Copenhagen and Milan to gather information on hereditary diseases in humans (Goldschmidt 1954). Eugenics, now perceived as a highly controversial political issue, was seen in the first half of the 20th century as an objective scientific discipline. In the 1950s, eugenics was broad, and encompassed—together with what we now refer to as eugenics—human genetics and genetic counseling (Kevles 1985).

Several years later, Goldschmidt expressed outright frustration at eugenics’ limited ability to bring long-term benefits to a population, and she concentrated on genetic counseling efforts intended to prevent births of babies suffering from serious, debilitating congenital conditions (Goldschmidt 1958). At this point, Goldschmidt was able to carry out her research on *Drosophila* as well as on humans, as she now headed a research team and no longer worked on her own. Members of the team were for the most part her young students, but also several physicians and nurses. Twenty years after she had been forced to give up her dream of being a physician, she had come full circle.^29^

In 1958, Goldschmidt became chair of the Genetics Society of Israel, which had a membership of sixty-four.^30^ About a third of the members in the Genetics Society of Israel were Goldschmidt’s students and wrote their graduate theses or doctoral dissertations under her supervision. As chair, in 1961 she organized an international conference in Jerusalem on the genetics of human populations. She invested great effort and exhibited organizational capabilities in fundraising and in getting many scientists, some world renowned, to participate. Her deputy in organizing the conference was Chaim Sheba, the director of Tel-Hashomer hospital. An NIH grant allowed twenty-four American geneticists and physicians to attend the conference and give a lecture (Goldschmidt 1963). Among them were Curt Stern and James Neel, two of the world’s leading human geneticists at that time. All in all, the conference attracted forty-three researchers from overseas, alongside one hundred Israeli participants.

With the successful 1961 international conference the field of genetics in Israel reached its first pinnacle. However, the feeling of success and satisfaction Goldschmidt probably felt after the conference was soon replaced with frustration over not being promoted to full professor. Goldschmidt’s career came to fruition and indeed peaked during a time of great change at the university. The State of Israel was established in 1948, and during the first years of Israeli statehood the number of students in the faculty of science grew rapidly.^31^ In fact, student numbers grew in all existing faculties, and as new study circles and departments in existing faculties formed, new faculties were also established. Disconnected from the rest of the city, from 1948 onwards the Mount Scopus campus no longer hosted classes, which instead took place in various locations throughout Jerusalem.^32^ While this situation caused hardship, it also led to a decentralization that made the system more dynamic and open to change. The changes in the university opened opportunities to all faculty members but especially to women who, at the time, faced greater obstacles than their male counterparts. For Goldschmidt, who had not been promoted since she began working for the university in 1944, this presented a chance for change. She became an instructor in 1949, a lecturer in 1954, and an associate professor in 1957.^33^ In April 1960, Goldschmidt was appointed Deputy Head of the Department of Zoology.^34^ At the end of the same year, she was elected to the University Senate.

Goldschmidt’s academic progress was halted when the university began stabilizing again; the number of students became constant, and the scattered facilities were relocated to the new campus at Givat Ram. This is consistent with the familiar pattern whereby women gained recognition within their professional sphere during periods of change and instability. For example, four women Hebrew poets gained recognition in Mandatory Palestine within just two years (1920–1922) as the result of the emigration of Hayim Nahman Bialik and other senior Hebrew writers and poets from the Soviet Union in the early 1920s. Their immigration left East Europe without any reputable Hebrew writers and poets. At that time, a stable literary center had not yet formed in Mandatory Palestine, and thus, a window of opportunity appeared for the women Hebrew poets (Miron 1991).

The procedures for promoting Goldschmidt to full professorship began in 1962. The proposal was discussed in the Appointment Committee in March 1963, but failed to achieve the necessary majority.^35^ When the proposal was brought up again three years later, once again the required majority was not achieved.^36^ Promotion at the university required more than being a qualified researcher and teacher and making a mark at the university—power struggles, cultural traditions and stereotypes also played parts. It is difficult to determine the degree to which the decision was influenced by the fact that the candidate was a woman and the decision makers were men. Nonetheless, research has shown that in considerations of promotion, women candidates experience gender discrimination. There is a tendency to express understanding toward a man’s success and suspicion toward that of a woman. The fact that women were not (and are still often not) considered to be the main breadwinners also might have worked against her. Women in academia were often seen as aliens, as not belonging, and as somewhat unreliable because of their supposed dual loyalty to home and career (Toren 2000; Wanneras and Wold 1997).

This was not the only case where advancement at the Hebrew University was difficult and painful, but Goldschmidt reacted differently than her male colleagues who had the same experience. Although she was insulted and hurt, Goldschmidt did not rebel actively, nor did she threaten to resign as did her male counterparts.^37^ In all her years at the university, Goldschmidt never used strong language toward her superiors. The only time she showed a more outspoken side was in her opposition against holding the international genetics conference in Germany in 1963, less than two decades after World War II and the Holocaust.^38^ Nevertheless, this was an ideological struggle, not a personal one. The repeated delays in her promotion upset Goldschmidt to the point that her husband wrote a personal letter to the Rector, without her knowledge. Finally, a year later, in December 1967, she received a favorable vote; she was promoted to the rank of full Professor.^39^

Another source of frustration for Goldschmidt was her failure to create an independent Department of Genetics. Up to the early 1950s, genetics research in Jerusalem functioned primarily as an auxiliary tool for research in taxonomy and evolution, congruent with the German understanding that genetics relates to the broader questions of development and evolution (Harwood 1993). This wide-ranging and holistic perception implied that genetics was relevant to older realms of scientific research; yet it did not allow the field to gain an independent definition and status. As American influence grew in the university, so did the awareness of the importance and necessity of establishing genetics as a discipline in its own right. In early 1963, Goldschmidt approached the Rector and Acting President of the University and asked him to separate genetics from zoology and create an independent department. At the next meeting of the Permanent Committee, it was mentioned that “should a suitable candidate [to head the department] be found, it would be desirable to set up a Department of Genetics.”^40^ It would seem, then, that Professor Elisabeth Goldschmidt— author of dozens of papers quoted by colleagues in Israel and overseas; a scientist who in many ways was the pioneer geneticist in Israel; the first chair of the genetics circle at Hebrew University; the first chair of the Genetics Society of Israel; and the author of the entry “genetics” in the Hebrew Encyclopedia—was not considered a suitable candidate to head a department.

Goldschmidt continued her academic activity, teaching and conducting research, between 1962 and 1970. She continued publishing articles at about the same pace as before, although usually not as sole author. After much hesitation, Goldschmidt decided in her final years to focus almost exclusively on human genetics (Wahrman 1972), and of her scientific publications between 1964 and 1970, only three articles were about *Drosophila* genetics, all the rest were on human genetics. Goldschmidt kept on learning and updating her knowledge. She had a sabbatical planned from August 1970 to July 1971, which she was going to divide between Cambridge, Rome, and the WHO (World Health Organization) in Geneva for studies and research in biochemical genetics and biometrics of human populations.^41^

However, it was a difficult period, and Goldschmidt was unable to shoulder the great stress imposed by her demanding career. An added difficulty was the election of her husband to the Knesset, the Israeli parliament, and his appointment as Deputy Minister of the Interior. It seems that Elisabeth Goldschmidt found it hard to cope with a situation in which her husband, Joseph, who had always provided her with endless support, was less available to do so.^42^ It is possible that the sabbatical year was planned in order to get away from the difficulties she faced in the Hebrew University. Goldschmidt, however, never went on sabbatical. While her students were out collecting flies, she committed suicide in her lab on May 6, 1970, at the age of fifty-seven.

To understand this event in a wider context, we need to consider the general picture of suicide among women in science, and recognize that this example was not an isolated case. Research has shown that women in science have a higher incidence of suicide, with the rates climbing higher among women who hold academic positions rather than those working in industry. For example, there is a five-fold increase of mortality by suicide among female members of the American Chemical Society as compared to the general figures of other American women (the rate for men of the ACS is double that for men in the United States overall). A correlation has also been found linking isolation, the leading work-related factor, and suicide (Walrath et al. 1985; Seiden and Gleiser 1990).

## Early Obstacles versus Late Obstacles

**Fig. 1:**
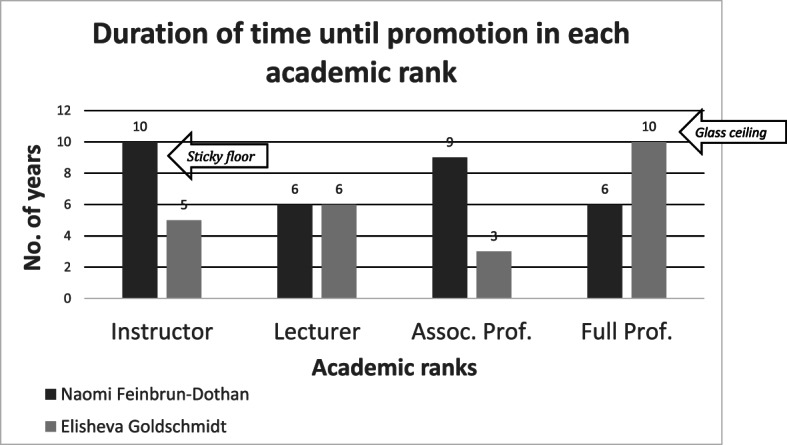
Duration of time until promotion in each academic rank

The transition from the rank of instructor to that of a lecturer lasted six years for both Feinbrun and Goldschmidt (see Fig. 1). Nevertheless, for the other three ranks, there were considerable differences between the two in the time staying in rank and before promotion. Goldschmidt experienced difficulty in the last step of the academic ladder. It took ten years from the time she received the title of associate professor until she was awarded the title of full professor; for Feinbrun the promotion to full professor took only six years. The longest stages for Feinbrun were getting to the rank of instructor (ten years) and then of associate professor (nine years). For Goldschmidt these promotions took five years and three years, respectively.

I will not delve into the gap of nine years between Feinbrun’s rank as lecturer and associate professor, mainly because neither she nor her colleagues regarded this as an abnormal situation. It is possible that no request was submitted for her promotion, and perhaps her academic achievements during the 1950s did not justify promotion to the rank of associate professor. I will focus on the period when each of the two researchers experienced a prolonged delay that was perceived as unjustified and caused great frustration. For Feinbrun it was making the first rank on the academic ladder, namely instructor, and for Goldschmidt it was the transition to the last step on this ladder, making the rank of full professor. How can these delays be explained?

Feinbrun became instructor after ten years, although in practice she fulfilled the tasks of this position much earlier. This was despite repeated demands from her department’s colleagues to do her justice. The reason for this was probably partly related to the good relations between the members of the botany department during the 1930s and 1940s, and the scientific collaborations between them. A significant portion of Feinbrun’s research was carried out as part of a team. It seems that as a young woman scientist she was seen as being first in the shadow of Alexander Eig, and after he passed away, in the shadows of Michael Zohary and Michael Evenari.^43^ It was of course convenient for the university management to employ Feinbrun for many years as an instructor de facto without giving her the rank and payment for it. Tellingly, most of the correspondence as to whether Feinbrun should be promoted to the rank of instructor was with the university’s treasurers.

During the 1960s, Feinbrun, who at the beginning of her career experienced a long delay, was promoted relatively quickly from the rank of associate professor to the rank of full professor. This was probably due, not only to the academic achievements she accumulated, but also the fact that she was not seen as a threat by her colleagues, especially since she received this rank at the age of sixty-six, two years before her retirement.

Goldschmidt did not encounter a barrier at the beginning of her career, and this was probably due to two main reasons. The first was her choice to focus on genetics. Being the only expert in an up-and-coming field enabled Goldschmidt to find her place at the Hebrew University. This is in accord with the well-known tendency of women toward peripheral and new scientific fields (Rentetzi 2004; Rayner-Canham and Rayner-Canham 1996). The second reason was the period of changes and decentralization at The Hebrew University in the years after the State of Israel was established in 1948, during which there was a huge increase in students’ numbers and new disciplines and fields were developed. Goldschmidt’s rapid advancement occurred between the years 1948 and 1961, a period of accelerated growth and decentralization at the university, and was halted thereafter, when the Hebrew University entered a period of stability.

Moreover, while Feinbrun was not seen as a threat to the gender hierarchy, Goldschmidt apparently challenged the existing gender and disciplinary hierarchies at the Hebrew University, as the growing prominence of genetics in the 1960s brought her from the margins into a central academic role. This may explain why members of the permanent committee refused to recognize her as someone who deserved to head a genetics department, and the nomination committee did not assume she deserved to rise to the highest rank on the academic ladder - the rank of full professor. The confidentiality of the nomination committee’s discussion and the fact that, to decide on promotion, at least two-thirds of the members had to vote in favor, made it easy to deny promotion. On two different occasions, no one voted against Goldschmidt’s promotion, but a number of committee members abstained. Thus, in contrast to her progress in the early stages which was at a reasonable pace, in the last stage she had to overcome serious career obstacles.

Throughout history, women in the sciences have often been distinguished from their male colleagues by the term “lady.” While this label was frequently used derogatorily, it was embraced with pride by many women. So-called lady scientists often had to navigate systemic barriers and find alternative ways to conduct their research, adapting to the challenges that sought to exclude them from the field (Phillips 1990). Naomi Feinbrun did not aspire to head an academic department or a scientific society. When she wrote about her scientific activity, she emphasized that she was part of a scientific team and focused more on her cooperation with her male colleagues, and less on her independent achievements (Ashbel 1991). I argue that Feinbrun, who operated according to the “lady scientist” model, was highly liable to experience early obstacles, since she seemed less qualified for a scientific career. Yet, if a woman like Feinbrun somehow managed to develop an academic career, she met with less opposition on the higher rungs of the academic ladder. The promotion of such a woman at a later stage might be relatively easy because she would not be perceived as a threat to her male colleagues.

On the other hand, a scientist who operated according to the ambitious and competitive male model may not have had a very challenging beginning but probably encountered a barrier at an advanced stage of her career.^44^ Thus, Goldschmidt, who was Israel’s greatest expert in the field of genetics during the 1950s and 1960s, who established and headed the genetics studies circle at The Hebrew University of Jerusalem, and who was the first chair of the Genetics Society of Israel, was seen by her male colleagues as a threat.

The question that now remains is—why did Feinbrun operate as a so-called lady scientist and what made Goldschmidt a more competitive scientist? Feinbrun, as we saw, did not aspire to an academic career; she was drawn to this path thanks to her meeting with Eig. Although she completed her undergraduate degree at the age of twenty-three, it took her a long time to return to academic studies and she completed her doctorate only at the age of thirty-eight. In comparison, Goldschmidt was very ambitious, and even though she was forced to emigrate twice—from Frankfurt to London, and from London to Jerusalem—following the rise of the Nazis, she still finished her doctoral studies at the age of thirty.

Apart from personality differences, these two scientists had different geographical and cultural backgrounds, and grew up in slightly different times. Goldschmidt was born and raised in Frankfurt-am-Main, Germany, which was home to Germany’s second-largest Jewish community, after Berlin. By 1925, Jews constituted 6.3% of the city’s population. The Jewish community in Frankfurt had a range of institutions, including two hospitals, four schools, an orphanage, and two cemeteries. In 1928, for instance, the first female Jewish judge in Germany was appointed in Frankfurt. It is likely that Goldschmidt, who was sixteen at the time, heard about the appointment and this strengthened her conviction that as a young Jewish woman the path to success and achievement was open to her as well. Feinbrun, who grew up in east Europe and was born twelve years earlier, was probably less exposed to the professional options open to women. In addition, differences between the Hebrew University’s botany and zoology departments may also have influenced their divergent career paths.

In 1933, the Hebrew University’s board of trustees appointed a committee to investigate the state of the university. The committee, chaired by Sir Philip Joseph Hartog, submitted a report in 1934 that severely criticized what was happening within the university. Among other things, the report described the dismal state of the zoology department, which had a depressing atmosphere and low efficiency. The report noted that the organizational and teaching skills of Shimon Fritz Bodenheimer, the head of the department, were meagre, and argued that he was not suitable for his position and called on him to resign as head of the department (Hartog 1934). The situation in the Department of Botany, by contrast, was presented in a positive light. The report described a small but talented team that had a fruitful cooperative relationship (Hartog 1934). The two departments’ characters did not change during the 1940s and 1950s.

The cooperative ethos of the Department of Botany contributed significantly to scientific achievements, but it did not necessarily translate into recognition of Feinbrun’s individual merit, particularly in the early stages of her academic career. As Margaret Rossiter has shown through her formulation of the “Matilda Effect,” women’s contributions to science have frequently been downplayed or attributed to their male colleagues (Rossiter 1993). In this context, it is likely that the university administration credited much of the department’s success primarily to Feinbrun’s male counterparts, which may have contributed to the delay in her academic advancement.

Goldschmidt was a PhD student and later an employee in a conflicted department, where some staff members did not talk to each other for years. Despite the unpleasant atmosphere however, it was precisely in such a place that Goldschmidt could conduct independent research and teaching at an early stage of her career, and her abilities could come to light.

## Conclusion

As Sharon Geva has argued, the tenure of Golda Meir as Israel’s Prime Minister (1969–1974) is frequently cited to reinforce the myth of gender equality in Israeli society (Geva 2020). The kibbutz movement, with its foundational principles of communal living and professed egalitarianism, along with Israel’s mandatory military service for both men and women, are often presented as evidence of equal treatment between the sexes. However, this narrative obscures a more complex reality. Meir was not directly elected by the public, but rather assumed office following the sudden death of Prime Minister Levi Eshkol. Similarly, while women in the kibbutzim were initially encouraged to participate in communal labor, they were often relegated to traditionally feminine roles such as teaching, kitchen work, and laundry. Israel’s army also exemplified this dynamic, with women subject to a patriarchal structure that confined them to subordinate positions. This tension between the rhetoric of equality and the persistence of gendered hierarchies was mirrored in other institutions, including universities. The disjunction between myth and reality underscores the limitations of the equality narrative in Israel and highlights the importance of critically examining how broader social structures shaped institutional attitudes toward women, particularly in academic settings.

Nina Toren, who has studied the professional careers of women in Israeli universities, has also emphasized the persistent gap between the widely held myth of gender equality—associated with the kibbutz, the military, and Israeli society more broadly—and the reality of entrenched traditional gender roles within both the family and the workplace (Toren 2000, 2005). According to Toren, powerful conservative social forces, combined with Israel’s pronatalist orientation, have created significant challenges for women seeking to build demanding professional careers. Her research shows that even in the final decades of the 20th century, all Israeli universities exhibited a striking contradiction between proclaimed commitments to gender equality and the persistent inequality experienced by women on the academic faculty. The challenges encountered by Feinbrun and Goldschmidt at the Hebrew University between the 1930s and the 1960s appear to prefigure those faced by many women in Israeli academia even decades later.

Toren analyzes the more “subtle” mechanisms of discrimination that shape women’s academic careers, focusing on the differing pace of advancement between men and women—a disparity rooted in what she terms a gendered timetable embedded within the organizational culture of academic institutions. These institutionalized expectations become self-fulfilling: women are presumed to advance more slowly, and structures evolve to reinforce that presumption. Among the methods used to enforce differentiated timetables are harsher evaluations of women’s academic achievements, bureaucratic delays, and the routine appointment of all-male nomination committees. The present article illustrates how such dynamics were already at work in earlier decades and highlights the variation in how gendered delays in promotion manifested in the cases of different women academics.

Feinbrun and Goldschmidt developed their scientific careers at the same institute, during the same period, and were active in two different sub-disciplines of biology. Yet, they encountered different kinds of hurdles – while Feinbrun had to deal with a continuous delay in her promotion at the beginning of her career, from the mid-1930s to the mid-1940s, Goldschmidt came across a barrier at the stage of advancement to professorship during the 1960s. Both succeeded in coping within a competitive and demanding environment that had a definitively masculine ethos, but there was a significant price for this coping. Their life stories may shed light on the varieties of challenges women scientists have faced in the past and are still facing today.

## Electronic Supplementary Material

Below is the link to the electronic supplementary material.

Supplementary Material 1

## Data Availability

No datasets were generated or analysed during the current study.

## Footnotes

1 The use of the category *Full Professor* in this article follows the formal academic ranking system in Israel, which consists of four ranks: Lecturer, Senior Lecturer, Associate Professor, and Full Professor. In Hebrew, these are: *Martzeh*, *Martzeh Bakhir*, *Professor Ḥaver*, and *Professor Min HaMinyan*. The highest rank, *Professor Min HaMinyan*, literally means “full-fledged professor,” although it is always translated as *Full Professor*. During the Hebrew University’s first decades there were four ranks but the first two ranks were Instructor (In Hebrew *Madrich*) and Lecturer while the two other were the same as today.

2 For more information, see The Israel Prize site, https://israel-prize.education.gov.il/israel-prize-recipients/tashnab-tashmag/#p_d85b717c-47eb-4401-93c7-0c8286bd2ba3 [Hebrew], accessed October 6, 2024. “Land of Israel” studies refers to an interdisciplinary approach to various research aspects about the Land of Israel. It includes different research fields, such as geography, botany, history, archaeology and others.

3 Marilyn Loden, “100 women: ‘Why I invented the glass ceiling phrase’”, December 13, 2017, *BBC News* (https://www.bbc.com/news/world-42026266, accessed October 6, 2024).

4 She later added “Dothan” to her last name, when her brothers Hebraized their family name.

5 Kirsh 2009. Naomi Feinbrun-Dothan. *The Jewish Women’s Archive Encyclopedia*, https://jwa.org/encyclopedia/article/feinbrun-dotan-naomi (accessed October 6, 2024).

6 Prof. N. Feinbrun-Dothan’s Curriculum Vitae, November 1965. Feinbrun personnel file, Hebrew University of Jerusalem Archives, Mount Scopus campus. Hereafter abbreviated “HUA-Feinbrun.”

7 The journal later became the *Israel Journal of Botany.*

8 “Travels and Research Tours” [no date] HUA, Evenari’s personnel file (until 1979). From 1921 to 1946, the Emirate of Transjordan was a British protectorate, so that Palestine and Transjordan were partially under the same governing power. Joseph Ben-David to whom it may concern, July 8, 1942, HUA 235/42; Ben-David to whom it may concern, July 14, 1942, HUA 235/42; Ben-David to the Aliya Department of the Mandatory Palestine, March 28, 1943, HUA 235/43; Michael Zohari, Michael Evenari, Tscharna Rayss, Naomi Feinbrun, and David Zaitschek to Michael Fekete, May 1940, HUA Botany Department File 235/40.

9 Dr. Naomi Feinbrun, “Curriculum Vitae,” June 3, 1957. HUA-Feinbrun.

10 Eig to Shlomo Ginzberg, December 3, 1936, HUA-Feinbrun.

11 Michael Evenari, Michael Zohary and Tscharna Rayss to the University Management, October 31, 1938, HUA-Feinbrun.

12 Evenari to the University Management, June 6, 1944, HUA-Feinbrun.

13 Andor Fodor to L.A. Mayer, November 27, 1944 HUA-Feinbrun.

14 Material for the Meeting of the Mathematics and Natural Sciences Faculty, April 23, 1945 HUA-Feinbrun.

15 Dr. Naomi Feinbrun, “Life Course,” [n.d.] HUA-Feinbrun.

16 Lists of the Academic Staff of the Hebrew University 1956, 1961, The National Library of Israel, Jerusalem.

17 Elisheva Goldschmidt, Curriculum Vitae, [n.d.]. Goldschmidt personnel file, HUA (hereafter “HUA-Goldschmidt”); Interview with Eliezer Goldschmidt (Elisheva’s son) by the author, July 23, 2002, Rehovot. Throughout her life, Goldschmidt used the name Elisheva in Hebrew and Elisabeth in English. In this article, I will use both names interchangeably.

18 Interview with Yemima Ben-Menahem (Elisheva’s daughter) by the author, August 21, 2002, Jerusalem.

19 Another woman who immigrated to Jerusalem in the 1930s, obtained her PhD under the supervision of Haas, and later had a successful scientific career as a developmental endocrinologist, is Haya Moscona Sobel (Passariello 2021).

20 Interviews with Goldschmidt’s students by the author: Elisheva Barak, February 26, 1998 and Giora Simchen, February 26, 1998, Jerusalem.

21 Interview with Elisheva Barak, February 26, 1998, Jerusalem.

22 Committee on zoology protocol, January 3, 1948; Report of the Committee on Zoology, November 17, 1948, both in HUA, Zoology Department File 236/48.

23 Georg Haas to Oskar Theodor, February 16, 1950, HUA-Goldschmidt.

24 High Salpeter to Goldschmidt, March 22, 1949, HUA-Goldschmidt.

25 Evenari, for example, always traveled with his wife (Evenari 1995).

26 Goldschmidt to Theodosius Dobzhansky, May 4, 1950, File B-D65: Theodosius Dobzhansky, The American Philosophical Society Library, Philadelphia, (hereafter: APS); Dobzhansky to Goldschmidt, May 12, 1950, HUA-Goldschmidt.

27 A number of emigré geneticists were located in Berkeley, and Curt Stern himself was, similar to Elisabeth Goldschmidt, a German Jewish immigrant (Smocovitis 2011).

28 The state of genetics in Jerusalem was influenced by its state in Germany and was changed only after Goldschmidt came back from the United States. On the two different traditions see Harwood (1993) for more details on the German system.

29 Her interest in genetic diseases and consanguineous marriage can be explained by her personal attraction to medicine, but also as part of her Zionist ideology and the huge waves of immigration (Kirsh 2003).

30 List of Genetics Society of Israel members [n.d.], The Genetics Society of Israel Archives, The Genetics Department of the Hebrew University, Givat Ram Campus (hereafter: GSIA).

31 *The Hebrew University of Jerusalem 1957*; *The Hebrew University of Jerusalem 1960*, Booklets, located at The National Library of Israel, Jerusalem.

32 *The Hebrew University of Jerusalem 1957*, Booklet, located at The National Library of Israel, Jerusalem.

33 Curriculum Vitae, Elisabet Goldschmidt, HUA-Goldschmidt.

34 Goldschmidt to Alexander, January 31, 1960; Mazar to Goldschmidt, June 5, 1960, HUA-Goldschmidt.

35 Five were in favor of this, three abstained; a positive decision required two-thirds of a positive vote. “Protocol of the nomination committee meeting no. 188,” March 3, 1963, HUA-Goldschmidt.

36 This time, four were favorable, but five abstained. “Protocol of the nomination committee meeting no. 249,” May 22, 1963, HUA-Goldschmidt.

37 Bodenheimer to the Dean of the Sciences and Mathematics Faculty, May 6, 1951, HUA 236/51; Reich to Mazar, May 2, 1955 HUA 236/55; Reich to Alexander, November 26, 1959, HUA 236/59.

38 Goldschmidt to Lerner, February 21, 1960, GSIA.

39 Joseph Goldshmidt to Rotenshtreich, June 8, 1966; “Protocol of the nomination committee,” December 17, 1967, HUA-Goldschmidt. There were eight votes in favor, and two abstentions.

40 Protocols of the permanent committee August 8, 1963, HUA, 232/48.

41 A request for sabbatical, February 2, 1970, HUA-Goldschmidt.

42 Interview with their son, Eliezer Goldschmidt, August 8, 1996, Rehovot.

43 It is a well-known phenomenon that the joint work of a man and a woman is attributed to the man (Rossiter 1993; Kirsh and Green 2021; Harvey 2012).

44 On the opportunities and barriers to women’s career in natural science and women’s different strategies to participation in scientific institutions see Madsen-Brooks (2009).
