# Supplementary material for: The Different Career Patterns of Two Pathbreaking Women Biologists at the Hebrew University of Jerusalem
Source: J Hist Biol. 2025 Sep 17;58(3):367–86. doi: 10.1007/s10739-025-09827-6 (PMC12657568; doi:10.1007/s10739-025-09827-6)
Supplement: Supplementary file 1 — Supplementary Material 1 [file 10739_2025_9827_MOESM1_ESM.docx]

Sticky floor G

Glass ceiling
